# Pain Management in Mandibular Molars With Symptomatic Irreversible Pulpitis: Mechanisms, Clinical Efficacy and Current Evidence: A Narrative Review

**DOI:** 10.1155/prm/8752885

**Published:** 2026-03-09

**Authors:** S. Eldafrawi, T. Elsewify, B. Eid

**Affiliations:** ^1^ Restorative Dental Sciences Department, College of Dentistry, Gulf Medical University, Ajman, UAE, gmu.ac.ae; ^2^ Endodontic Department, Faculty of Dentistry, Ain Shams University, Cairo, Egypt, asu.edu.eg

**Keywords:** cryotherapy, endodontic pain, intrapulpal anaesthesia, local anaesthesia, nerve block failure, supplemental anaesthesia, symptomatic irreversible pulpitis

## Abstract

**Background:**

Pain control remains a cornerstone in endodontic practice, particularly in cases of symptomatic irreversible pulpitis where achieving profound anaesthesia is often challenging. As the inferior alveolar nerve block (IANB) frequently fails to provide profound pulpal anaesthesia, optimizing pain control in such cases requires evidence‐based modifications to anaesthetic techniques and the use of supplemental approaches.

**Aim:**

This narrative review aims to synthesize current evidence on clinical strategies for improving pain management in mandibular molars with symptomatic irreversible pulpitis, with particular emphasis on enhancing the efficacy of IANB and supplemental injection techniques.

**Methods:**

A narrative review methodology was employed to synthesize clinical trials and systematic reviews published between 1975 and 2025.

**Results:**

Evidence indicates that increasing the anaesthetic volume and applying supplemental techniques, including intraligamentary, intraosseous and intrapulpal injections, significantly enhances anaesthesia success. Adjunctive measures such as cryotherapy and temperature modification of anaesthetic solutions further improve pain control. The integration of these methods provides a predictable and multimodal approach to managing endodontic pain in challenging clinical scenarios.

**Conclusion:**

Effective pain management in mandibular molars with symptomatic irreversible pulpitis relies on a comprehensive strategy that combines optimized anaesthetic volume and supplemental techniques. Intrapulpal anaesthesia remains essential for achieving pulpal anaesthesia when all other techniques fail. Continued clinical research is essential to refine these approaches and establish standardized protocols for achieving reliable anaesthesia in endodontic practice.

## 1. Introduction

Symptomatic irreversible pulpitis is defined as the inflammation of the pulp as a response to injurious stimuli and is characterized by pain that is severe in nature [1]. In order to alleviate the symptoms and eliminate the infection, a root canal treatment (RCT) is necessary [1, 2]. The anaesthetic technique of choice for mandibular teeth is the inferior alveolar nerve block (IANB) [3]. This technique, however, demonstrates a failure rate of 44%–81% in teeth with symptomatic irreversible pulpitis [4]. Therefore, there exist supplementary anaesthetic techniques that are used in conjunction with nerve blocks that aim to reduce or eliminate intraoperative pain. Supplementary anaesthesia could be administered within the periodontal ligament (intraligamentary), within the buccal vestibule (buccal infiltration), within the surrounding alveolar bone (intraosseous) or within the canal space itself (intrapulpal) [5]. Cryoanaesthesia, or altering the anaesthetic solution temperature, might also be employed to enhance the pain management [6].

## 2. Methodology

A narrative review methodology was adopted to comprehensively synthesize and critically evaluate the existing literature on the mechanisms, clinical efficacy and current evidence pertaining to pain management in symptomatic irreversible pulpitis. An extensive electronic search was conducted across the Scopus, Web of Science, ScienceDirect, Cochrane, PubMed and Google Scholar databases to identify relevant publications from 1975 to 2025. The following keywords were used interchangeably on all databases: symptomatic irreversible pulpitis, mandibular molars, IANB, supplementary anaesthesia, cryotherapy and success rate. Eligible studies included clinical trials and systematic reviews directly addressing pain management approaches in cases of symptomatic irreversible pulpitis in mandibular molars. Exclusion criteria comprised articles not published in English, case reports, conference abstracts and studies unrelated to endodontic pain management.

### 2.1. Success Rate of Primary Anaesthetic Techniques in Symptomatic Irreversible Pulpitis

Certain hurdles may present to the clinician regarding the IANB, such as anaesthetic failure, noncontinuous anaesthesia, delayed onset and limited duration of action. These complications can be seen in 43%–57% of cases of noninflamed mandibular molars following an IANB [7]. In cases where the tooth in question has an inflamed pulp, achieving adequate anaesthesia becomes a greater challenge. In addition to the dense cortical bone, limited anaesthetic diffusion and anatomical variations in mandibular innervation, cases of SIP, where inflammatory mediators lower the activation threshold of nociceptors and alter nerve excitability, show a greater challenge. Despite multiple proposed explanations, the justification for inadequate pulpal anaesthesia in SIP remains incomplete. One widely cited hypothesis is the sodium channel theory, which suggests an upregulation of tetrodotoxin‐resistant sodium channels in inflamed pulp tissue, rendering local anaesthetics less effective at blocking nerve conduction. The inflamed status of the pulp, whether symptomatic or asymptomatic, plays a role in decreasing the pain threshold, making the tooth more sensitive to a lower stimulus than that of an uninflamed pulp. This phenomenon can be attributed to the hyperalgesia theory, which links the presence of inflammation with alterations along the nerves, thus leading to more significant reactivity to lower stimuli [8]. In addition to alterations along the nerves, inflammation also has a direct role in lowering the pH of the associated local tissue. This results in inflammation‐induced acidosis, therefore preventing the anaesthetic from shifting from its ionized acid form to its nonionized base form, which is crucial for anaesthetic diffusion across the cell membrane [9]. It is also important to note that even when the IAN is blocked, the mylohyoid nerve provides mandibular molars with associated innervation in 10%–20% of cases, making complete pulpal anaesthesia more challenging to achieve [10].

Traditional methods of confirming anaesthetic success include questioning the patient on the presence of subjective indicators, such as soft tissue numbness. In symptomatic vital cases, however, this is not always a clear indication of profound pulpal anaesthesia, and objective methods, such as a lack of response to a cold refrigerant or an electric pulp tester (EPT), are preferred [11].

The assessment of anaesthetic success in endodontic research is inherently heterogeneous, with studies adopting variable definitions such as absence of pain during access cavity preparation, reduction in intraoperative pain, responses to pulpal sensibility testing or subjective pain scoring systems. This variability reflects the multifactorial and clinically nuanced nature of anaesthetic success rather than a methodological shortcoming. However, to minimize conceptual ambiguity, precise definitional clarification is essential.

In this review, a distinction is made between objective measures of pulpal anaesthesia, defined as the demonstrable loss of pulpal neural responsiveness assessed through thermal or electrical sensibility testing, and subjective patient‐reported pain outcomes, which reflect the individual’s perceptual experience during operative procedures. Although interrelated, these constructs represent distinct physiological and experiential domains.

Presenting outcomes using the original definitions reported by individual studies ensures transparency; however, interpreting them within this structured framework enhances conceptual coherence and enables clearer synthesis of both biological and patient‐centred dimensions of anaesthetic evaluation in clinical endodontics [12].

There exist several approaches to enhancing the efficiency of pulpal anaesthesia. These approaches include utilizing alternative solutions, increasing the anaesthetic volume and concentration, using modified injection techniques, such as the Gow‐Gates and Vazirani‐Akinosi techniques, as well as the use of supplemental anaesthetics, including intraosseous, intraligamentary, intraosseous and intrapulpal injections, in addition to altering the anaesthetic solution temperature (cryoanaesthesia).

#### 2.1.1. Effect of Alternative Solutions on Pulpal Anaesthesia

The literature contains several studies [13–18], suggesting that different anaesthetic solutions, even when utilized for the same anaesthetic techniques, may achieve different levels of pulpal anaesthesia. This was explored in a randomized clinical study comparing the efficiency of 2 anaesthetic protocols for IANB: a combination of 3% mepivacaine and 2% lidocaine, and two cartridges of 2% lidocaine. The results revealed no statistically significant difference between the groups, while also noting a lack of difference regarding injection pain, as well as onset time [13].

A meta‐analysis concluded that articaine could be a superior anaesthetic solution to lidocaine for mandibular teeth diagnosed with symptomatic irreversible pulpitis [14]. To further support this conclusion, a high‐quality evidence systematic review encompassing the results of 16 studies was conducted, where articaine was found to have a significantly positive impact on increasing the success rate of IANB, raising it to 73%. In addition, it was also noted that among articaine, prilocaine, mepivacaine, bupivacaine and lidocaine, the latter demonstrated the weakest performance in IANB, with a success rate of 12% [15]. It is important to note, however, that the use of articaine has been linked with a potential increase in risk of paraesthesia, as stated by Hopman et al. [19] and Piccinni et al. [20].

An additional meta‐analysis also aimed to investigate whether anaesthetic solutions play a role in the success rate of IANB. A total of 11 studies met the inclusion criteria, and the following solutions were compared: mepivacaine, prilocaine, articaine, bupivacaine and lidocaine. While mepivacaine achieved the most profound pulpal anaesthesia, and lidocaine the least, the results failed to demonstrate a statistically significant difference. The quality of evidence for this meta‐analysis, however, was deemed low to moderate [16].

Similarly, the difference in IANB success rates between 2% mepivacaine and 4% articaine was evaluated via a randomized clinical trial. No significant difference was revealed as a result of this study, with mepivacaine and articaine showing success rates of 35.8% and 41.2%, respectively. Moreover, the frequency of supplemental anaesthesia administration was also noted, with 43.6% for mepivacaine and 38.2% for articaine. These results highlight the importance and need for utilizing supplemental anaesthesia when treating mandibular teeth diagnosed with symptomatic irreversible pulpitis [18]. In addition, a well‐designed randomized controlled trial comparing two anaesthetic solutions and gauging the intraoperative pain levels associated with each was performed. The study allocated one hundred and fifty‐two patients to two groups: one receiving 2% lidocaine and the other receiving 4% articaine. The results, however, were similar to those of previous literature concerning the same topic, where no significant difference was found between the two solutions [17].

In conclusion, the literature does not present any clear evidence of the superiority of one anaesthetic solution over the other in terms of success and efficiency of pulpal anaesthesia.

#### 2.1.2. Effect of Anaesthetic Volume and Concentration on Pulpal Anaesthesia

Alternatively, it has also been hypothesized that the volume of the anaesthetic, rather than the solution, has an effect on the profoundness of pulpal anaesthesia achieved via IANB. Higher volumes of the same anaesthetic are theorized to result in more efficient anaesthesia. This could be attributed to the fact that higher volumes fill the pterygomandibular space more than lower volumes, thus exposing more length of the nerve trunk to the anaesthetic solution [21]. This concept was explored in a randomized clinical trial involving 82 patients with mandibular first molars diagnosed with symptomatic irreversible pulpitis, half of whom received an IANB of 1.8 mL of 4% articaine with 1 : 100,000, while the second half received 3.6 mL of the same solution. IANB success rates were found to be 27.5% and 77.5%, respectively, implying that increasing the anaesthetic volume is linked with more profound anaesthesia [22]. Additionally, a systematic review involving five studies concluded that in cases of mandibular molars with irreversible pulpitis, increasing the volume does lead to higher chances of IANB success. The overall success rate, however, was still found to be unsatisfactory, where the utilization of supplemental techniques remains necessary in order to provide patients with a pain‐free experience [23]. Similarly, a systematic review was conducted where four clinical trials fit the selection criteria, all of which compared the success rates of IANB with the same solution, but in different volumes of either 3.6 mL or 1.8 mL. The results stated that using a higher volume of the same anaesthetic solution may increase the success rate by 36% [16].

In contrast, a randomized clinical trial comprising 90 patients with irreversibly inflamed mandibular molars revealed no statistically significant difference between a group receiving 1.8 mL of 4% articaine with 1:100,000 epinephrine and another receiving 3.6 mL of the same anaesthetic solution [24]. In corroboration of these results, Fowler and Reader [25] also cited no statistically significant difference in the success rate of an IANB using 1.8 mL or 3.6 mL of 2% lidocaine on a sample size of three hundred and nineteen patients diagnosed with symptomatic irreversible pulpitis.

While a general consensus is yet to be determined, the majority of the present literature has cited a significant increase in the profoundness of pulpal anaesthesia associated with utilizing a larger volume of the anaesthetic solution.

#### 2.1.3. Effect of Alternative Anaesthetic Techniques on Pulpal Anaesthesia

The utilization of alternative anaesthetic techniques, such as the Gow‐Gates technique, might achieve varying levels of anaesthesia [26]. One hundred and fifty patients diagnosed with symptomatic irreversible pulpitis in relation to a mandibular molar received either 2 Gow‐Gates injections, 2 IANB injections or 1 Gow‐Gates combined with 1 IANB, all of which contained 2% lidocaine with 1 : 80,000 epinephrine. The group receiving a combination of the two techniques exhibited a significantly higher anaesthetic success rate of 70% compared to the other groups. There was, however, a lack of a statistically significant difference between the Gow‐Gates group and the IANB. These results were further supported in randomized controlled trial that compared the efficacy of 2% lidocaine with 1 : 100,000 epinephrine when administered as a traditional IANB, versus as a Gow‐Gates injection, on 80 patients with irreversibly inflamed mandibular molars, citing no statistically significant difference between the two [27]. In contrast, a systematic review analysing the results of four studies reached the conclusion that the Gow‐Gates technique demonstrated higher anaesthetic efficiency in comparison to IANB. The literature included in this analysis, however, was found to be of fair to poor quality [10].

Additionally, the difference in anaesthetic efficacy between the Gow‐Gates, IANB and Vazirani‐Akinosi technique was evaluated on two hundred and ten patients requiring minor surgical procedures in the mandibular region. The only statistically significant difference detected was when comparing the Vazirani‐Akinosi technique to the two other groups, where the anaesthetic success was found to be 95.71% [28]. Moreover, a meta‐analysis comparing the anaesthetic efficacy of the Gow‐Gates technique, IANB and mental incisive nerve blocks in treating mandibular teeth diagnosed with irreversible pulpitis revealed no statistically significant difference between all groups, where the cumulative success rate was deemed low, thus highlighting the importance of supplementary anaesthesia [29].

As demonstrated by the aforementioned authors, the trend at hand appears to show a weak correlation between anaesthetic technique and the degree of pulpal anaesthesia achieved.

#### 2.1.4. Alterations to Anaesthetic Solution

In addition to the anaesthetic solution, volume and technique, the concept of altering the temperature and pH of the anaesthetic solution is thought to aid in achieving more profound pulpal anaesthesia. This hypothesis was investigated in a randomized clinical trial consisting of three groups of patients, all diagnosed with symptomatic irreversible pulpitis. In this study, one group received 2% lignocaine, one received 2% lignocaine solutions which were preheated to 42°C (administered at 37°C), and one received 2% lignocaine buffered with 0.18 mL of 8.4% sodium bicarbonate. Statistical analysis showed no significant difference in intraoperative pain reduction between the preheated group and the buffered group. A statistically significant difference, however, was discovered when comparing the buffered group to the control group, as well as the preheated group to the control group. Both experimental groups were more efficient in intraoperative pain reduction compared to the control group [30]. In contrast, no statistically significant difference was reported between 2% lidocaine nonbuffered and buffered with 8.4% sodium bicarbonate in a clinical trial conducted on 80 adult patients with irreversibly inflamed mandibular posterior teeth [31].

In addition to its effect on anaesthetic success, buffering the anaesthetic solution with 8.4% sodium bicarbonate may also influence other factors such as pain on injection and onset of anaesthesia. This was explored on one hundred patients, wherein 2% lignocaine was used to administer three nerve blocks: inferior alveolar, lingual and long buccal. Results revealed the buffered solution completely eliminated injection pain, as opposed to the 78% incidence of pain in the nonbuffered group. The buffered group also reported a significantly shorter time of onset of anaesthesia (34.4 s) compared to the nonbuffered group (109.76 s) [32].

### 2.2. Supplemental Anaesthesia

While the current literature references several methods of increasing the anaesthetic efficiency of IANB, the issue of intraoperative pain during endodontic treatment of mandibular molars diagnosed with symptomatic irreversible pulpitis persists. Therefore, supplemental anaesthetic techniques are of great importance, and their effect on pulpal anaesthesia in cases where IANB was proven insufficient has been largely covered within the literature.

#### 2.2.1. Infiltration

The majority of the literature concerning supplementary infiltration techniques seems to be focused on buccal infiltrations specifically, with a scarcity of studies investigating whether lingual infiltrations are a viable supplementary technique. Dou et al. [33] aimed to investigate whether administering a buccal and lingual infiltration as a supplement to IANB, rather than the traditional method of a buccal infiltration alone, would lead to more pronounced pulpal anaesthesia. Eighty patients diagnosed with irreversible pulpitis participated in this study, all of whom received a standard IANB of 4 mL 2% lidocaine supplemented by either a buccal infiltration of 0.9 mL 4% articaine or a combination of both lingual and buccal infiltrations of the same volume and solution. The results, however, did not display a statistically significant difference. Similarly, the success rates of buccal infiltrations as opposed to lingual infiltrations were compared, both following an unsuccessful IANB. Sixty patients with mandibular molars diagnosed with symptomatic irreversible pulpitis received an IANB using 4% lidocaine. Cases of a failed IANB received either a buccal or lingual infiltration, both of 1.8 mL 4% articaine. Results revealed a statistically significant difference concerning first molars, where a buccal infiltration was deemed more successful than lingual. In second molars, however, there was a lack of a statistically significant difference. It can therefore be assumed that the supplementary infiltration technique of choice remains the buccal infiltration [34].

A randomized study examined whether the use of different anaesthetic solutions as a supplementary buccal infiltration achieved different levels of pulpal anaesthesia. The solutions in question were 2% lidocaine and 4% articaine, both of which followed an unsuccessful IANB. Statistical analysis revealed that a buccal infiltration using 2% lidocaine was successful in only 29% of the time, while 4% articaine was successful in 71% of the time [35]. A similar randomized clinical trial wherein the sample was one hundred mandibular molars diagnosed with irreversible pulpitis was performed. The conclusion was also in favour of the use of 4% articaine as a supplemental buccal infiltration as opposed to 2% lidocaine. The success rate was found to be 62% for articaine and 37% for lidocaine, indicating a statistically significant difference between the two solutions [36]. The efficacy of 2% mepivacaine, compared with 4% articaine and 2% lidocaine as solutions for a supplementary buccal infiltration for mandibular posterior teeth with irreversible pulpitis was investigated [37]. Out of the 220 patients participating in the study, all of whom received an IANB of 2% lidocaine, 156 reported unsuccessful anaesthesia. These patients received a supplemental buccal infiltration of either 4% articaine, 2% lidocaine or 2% mepivacaine. Results revealed a statistically significant difference in the success rate of the articaine group, which was calculated to be 83.3%, in comparison to the lidocaine group and the mepivacaine group, where the success rates were 57.1% and 59.5%, respectively. As demonstrated by the present literature, 4% articaine appears to be the anaesthetic solution of choice for supplementary buccal infiltrations, wherein it exhibits a higher anaesthetic success rate than that of 2% lidocaine.

Further highlighting the importance of supplemental anaesthetic techniques in cases of mandibular molars diagnosed with symptomatic irreversible pulpitis, a systematic review analysed a total of 14 studies comparing the success rate of IANB alone and IANB in conjunction with supplemental infiltration. The overall success rate for IANB alone was 14%–39%, while that of IANB supplemented by a buccal infiltration was 50%–65%. While the difference was apparent clinically, statistical analysis stated no significant difference between the two techniques, thus emphasizing the importance of exploring alternative supplementary techniques, which may provide a higher incidence of profound anaesthesia [38].

In addition to the anaesthetic solution of choice, it is also important to consider the effect of the anaesthetic volume on the degree of anaesthesia obtained. This concept was studied where 1.8 mL and 3.6 mL of 4% articaine as a supplementary buccal infiltration were compared in terms of anaesthetic efficacy. Two hundred and thirty‐four patients with mandibular molars with irreversible pulpitis received a standard IANB of 4% articaine, where a success rate of 37% was noted. The cases with a failed IANB received either 1.8 mL or 3.6 mL or 4% articaine as a supplementary buccal infiltration, where the success rate was determined to be 62% and 64%, respectively. This leads to the conclusion that the volume of the solution does not have a statistically significant effect on the rate of anaesthetic efficiency [39].

#### 2.2.2. Intraosseous

Intraosseous anaesthesia is a different method used alongside IANB to enhance the degree of pulpal anaesthesia achieved. Intraosseous injections, however, require the use of special armamentarium in order to deliver the anaesthetic solution into the cancellous bone surrounding the tooth in question. Examples of his supplementary technique include the Stabident (Fairfax Dental Inc., Miami, Florida), QuickSleeper (Dental HiTec, Cholet, France) and X‐tip (Dentsply Sirona Inc., York, Pennsylvania, USA) systems, where the latter was explored in an *in vivo* study [40]. This system comprises a special hollow needle that acts as both a drill and a guide sleeve. The drill aids in allowing the guide sleeve to reach the cortical plate, after which it is separated and removed, leaving the guide sleeve embedded within the bone in order to deliver the solution. The removal of the embedded guide sleeve is done postinjection, with the aid of a haemostat. The solution of choice for this study was 1.7 mL of 4% articaine, administered to patients who required additional anaesthesia. Upon statistical analysis, it was discovered that 83.3% of the X‐tip injections administered were proven successful following inadequate pulpal anaesthesia via IANB. To further support the aforementioned results, a similar clinical study was conducted [41]. The study at hand also aimed to investigate the success rate of intraosseous anaesthesia administered via X‐tip as a supplement to an unsuccessful IANB, where 4% articaine was the solution used for both injections. Out of twenty‐four intraosseous injections administered secondary to IANB, 21 were considered successful, where the success rate was calculated to be 87.5%.

Moreover, the use of intraseptal supplementary anaesthesia was further investigated on one hundred patients with mandibular molars diagnosed with symptomatic irreversible pulpitis, all of whom received an IANB of 2% lidocaine, with a success rate of 25%. In cases of insufficient anaesthesia, an additional intraseptal injection of 0.7 mL 4% articaine, with the assistance of a computer‐controlled local anaesthetic delivery unit, was administered, where a success rate of 29% was seen. Statistical analysis revealed no significant difference between the two groups [42].

Intraosseous injections are associated with certain adverse effects that clinicians should consider. Transient increases in heart rate and blood pressure have been reported, likely due to the rapid systemic absorption of vasoconstrictor‐containing solutions [43]. Additionally, patients may experience localized postinjection discomfort, occasional soft tissue trauma or temporary injection site bleeding. Awareness of these potential side effects allows clinicians to balance anaesthetic efficacy with patient safety and to select appropriate supplemental techniques based on individual patient factors.

There remains a scarcity of research on the extent of pulpal anaesthesia achieved through supplementing an IANB with an intraosseous injection. While some authors [40, 41] have reported a significant increase in the anaesthetic success rate, others [42] reported the opposite, deeming this a controversial topic, where no final conclusion can be drawn.

#### 2.2.3. Intraligamentary

Intraligamentary injections are yet another form of supplemental anaesthesia often utilized as an adjunct to IANB. This anaesthetic technique is typically done using a short needle, which is placed within the gingival sulcus at a 30° angle and pushed as apically as possible. A volume of 0.2 mL is usually injected over the course of 20 s, under backpressure. This process is done on the mesial aspect of the tooth in question, after which it can be replicated on the distal side. While the onset of this technique is rapid, and usually reported within 30 s, the duration of action is limited to 10–45 min [44–46]. Several studies have been conducted to evaluate the potency and duration of the intraligament anaesthesia.

Parirokh et al. conducted a randomized controlled trial, wherein patients either received an IANB alone or IANB in combination with both a buccal infiltration and an intraligament injection. A statistically significant difference was discovered between both groups, with a success rate of 22% for the IANB group and 58% for the group receiving an IANB combined with a buccal infiltration and intraligamentary injection [47].

Alternatively, Lin et al. designed a retrospective study investigating whether the use of an intraligamentary injection as a primary anaesthetic technique would prove more successful than IANB. A total of one hundred and fifty mandibular molars diagnosed with asymptomatic irreversible pulpitis received an initial intraligamentary injection of 0.2 mL 4% articaine at the mesiobuccal and distobuccal aspects of the tooth to be treated. Reports of insufficient anaesthesia were followed by an additional intraligamentary injection at the mesiolingual and distolingual aspects of the tooth. The overall success of intraligamentary anaesthesia as a primary injection was reported to be 92.1%, out of which 31.8% were sufficiently anesthetized using the two‐site injection technique, and 60.3% required the four‐site technique to completely eliminate intraoperative pain. Anaesthetic failure leading to the need for an IANB was only reported in 7.9% of the cases [48].

Aggarwal et al. proposed a theory for improving the success rate of intraligamentary injections by increasing the volume of solution administered. In this randomized clinical trial, 97 patients diagnosed with symptomatic irreversible pulpitis first received an IANB of 2% lidocaine, followed by an intraligamentary injection of either 0.2 mL or 0.6 mL per root of 2% lidocaine, in cases where the IANB had failed. The results revealed a success rate of IANB in 20% of the cases, while the intraligamentary injections of 0.2 mL and 0.6 mL were successful in 64% and 84% of cases, respectively, thus concluding that increasing the volume of anaesthetic solution is linked to a significant increase in anaesthetic efficiency [49].

In addition to the volume of solution deposited, Aggarwal et al. later suggested that the use of different anaesthetic solutions might impact the efficacy of supplemental intraligamentary injections. One hundred and six patients with mandibular molars diagnosed with symptomatic irreversible pulpitis received an IANB of 2% lidocaine, out of which 19% were successful. Those who reported intraoperative pain received an intraligamentary injection of either 0.6 mL per root of 4% articaine or 2% lidocaine, where a statistically significant difference in success rates was not found [50]. Moreover, the effect of the different anaesthetic concentrations on the success of intraligamentary injections was also observed in a later study [51]. An IANB of 2% lidocaine with 1 : 80,000 epinephrine was administered to one hundred and eighteen patients with mandibular molars diagnosed with symptomatic irreversible pulpitis, out of which 23% were successful. Patients who reported unsuccessful anaesthesia were subjected to an intraligamentary injection of 0.6 mL per root of either 2% lidocaine with 1 : 80,000 epinephrine or 2% lidocaine with 1 : 200,000 epinephrine, which exhibited success rates of 82% and 57%, respectively.

A further study was carried out comparing the use of 2% lidocaine and 4% articaine in mandibular first and second molars with irreversible pulpitis. Seventy‐six patients were included in this study, half of whom received an IANB using 2% lidocaine, while the other half received 4% articaine, with an overall success rate of 13.5%. Those who reported EPT values lower than 100 were given a 0.2 mL per root intraligamentary injection of the same solution they received for the IANB, where 4% articaine was found to result in the most profound pulpal anaesthesia in mandibular second molars, exhibiting 100% success rate [52]. A similar study comparing the efficiency of intraligamentary injections using 2% lidocaine and 4% articaine was conducted, where the sample comprised one hundred and forty‐seven patients with mandibular molars diagnosed with both symptomatic irreversible pulpitis and symptomatic apical periodontitis. The IANB of 2% lidocaine displayed a success rate of less than 50%, wherein those reporting failure received an intraligamentary injection of either 2% lidocaine or 4% articaine. The results failed to display a significant difference between the two groups, indicating that anaesthetic solution plays a minimal role in anaesthetic success [53].

In regard to the use of intraligamentary anaesthesia, the literature supports the statement that its combination with an IANB is linked with a higher degree of pulpal anaesthesia. The question remains whether anaesthetic volume, solution and concentration lead to an even more significant increase in the anaesthetic success rate.

#### 2.2.4. Combination of Supplemental Anaesthetic Techniques

Due to a lack of consensus regarding the most effective method of supplementing an IANB to achieve maximum anaesthetic efficiency, Dianat et al. designed a randomized clinical trial with the aim of comparing multiple anaesthetic techniques and their efficacy concerning mandibular molars diagnosed with symptomatic irreversible pulpitis. This study comprised 90 patients and three groups: the first receiving an IANB of 2% lidocaine, another receiving an IANB with 2% lidocaine supplemented by a buccal infiltration of 4% articaine and another receiving an IANB of 2% lidocaine supplemented by both a buccal infiltration and an intraseptal injection, both using 4% articaine. The success rate for each group was 30.33%, 66.66% and 80%, respectively, with statistical significance found between all groups [54].

In a further attempt to determine whether a superior supplemental anaesthetic method exists regarding treating mandibular molars with symptomatic irreversible pulpitis, a randomized clinical trial was designed to compare three supplemental anaesthetic techniques: intraosseous, intraligamentary and buccal infiltration, all in conjunction with a standard IANB, and all using 4% articaine. The control group, which received an IANB without any supplementary method, exhibited a success rate of 40%. IANB, in conjunction with an intraosseous injection, however, showed the highest success rate of 92.5%, with a statistically significant difference when compared with all the groups [43].

A similar study aimed to investigate the difference in anaesthetic success between buccal infiltrations and intraligamentary injections, both using 4% articaine. This trial studied one hundred patients with mandibular first or second molars with irreversible pulpitis, where an initial IANB of 2% lidocaine was administered, followed by either an intraligamentary injection or a buccal infiltration. The overall results revealed a success rate of 80% for the intraligamentary group and 74% for the buccal infiltration group, citing no statistically significant difference. A significant difference was, however, noted in regard to mandibular second molars, where intraligamentary injection showed a success rate of 92% as opposed to 64% for the buccal infiltration group [55].

Moreover, different success rates of several supplementary anaesthetic techniques were compared, while also comparing different anaesthetic solutions, namely, articaine and mepivacaine. A sample of one hundred and twenty patients with mandibular first molars diagnosed with symptomatic irreversible pulpitis were selected, where an IANB of 2% lidocaine was administered prior to endodontic treatment. Those who reported subjective IANB symptoms, but failed to demonstrate pulpal anaesthesia upon EPT were then assigned to one of the following groups: Group 1 received a buccal infiltration of 4% articaine, Group 2 received a four‐site intraligamentary injection with 4% articaine, Group 3 received a buccal infiltration of 2% mepivacaine, and Group 4 received a four‐site intraligamentary injection with 2% mepivacaine. The success rate of each group was then concluded to be 90% for Group 1, 66.67% for Group 2, 70% for Group 3 and 50% for Group 4, where a statistically significant difference was noted between all groups. Moreover, the overall success rate of articaine and mepivacaine was calculated to be 78.33% and 60%, respectively, hence concluding that a combination of articaine and a buccal infiltration proved to achieve the most profound pulpal anaesthesia in cases of mandibular first molars with irreversible pulpitis [56].

A different approach for increasing anaesthetic success was conducted. In this study, 94 patients receiving the traditional IANB using 2% lidocaine were also subjected to nitrous oxide sedation. In cases of failure, which occurred in 56% of the sample, the patients were given an additional intraligamentary injection of 0.2 mL per root using 4% articaine. If the patient continued to experience intraoperative pain, they received a second intraligamentary injection following the same protocol as that of the first. Out of all patients participating in this study, 69% reported sufficient anaesthesia following a single intraligamentary injection, and the remainder of the sample required a second supplemental injection, where an 80% success rate was observed. Therefore, it was concluded that even with a combination of IANB, nitrous oxide and two sets of intraligamentary injections, complete pulpal anaesthesia was still not obtained [57].

#### 2.2.5. Intrapulpal Anaesthesia

In certain cases, despite the utilization of both IANB and supplemental anaesthesia in the form of infiltrations, intraligamentary or intraosseous injections, complete pulpal anaesthesia is yet to be achieved, and the incidence of intraoperative pain persists. This is seen in 5%–10% of mandibular posterior teeth diagnosed with irreversible pulpitis [58]. In such cases, intrapulpal injections may be put to use. Intrapulpal anaesthesia refers to a technique in which the anaesthetic solution is deposited directly into the pulp by wedging the needle either into an exposure in the pulp chamber or into the canal space itself. One of the major advantages of intrapulpal anaesthesia compared to other supplemental techniques is that it does not require removal of the rubber dam, thus reducing the risk of leakage throughout the procedure. This technique, however, relies heavily on the presence of back pressure during injection [5, 6, 59].

The resistance refers to the mechanical opposition created by the confined tissue environment or a sealed access, which enables the development of back pressure and the hydrostatic pressure responsible for achieving effective anaesthesia. To ensure back pressure, the size of the exposure during access cavity preparation should be kept as small as possible prior to injection, wherein the needle should be firmly wedged. If, however, the opening is too large, it is also possible to attempt to advance it within the canal space until a tight fit is obtained. Alternatively, the needle can also be surrounded by a piece of wax or gauze, which aids in obtaining the resistance necessary to perform the injection under sufficient pressure [60]. Typically, 0.2 mL of solution is administered [58, 61], and the duration of action is thought to be limited to 15–20 min [59, 61], during which the clinician should perform extirpation of the inflamed pulpal tissue.

The exact mechanism by which intrapulpal anaesthesia works remains uncertain. A recent literature review suggests two possible mechanisms: the first of which is direct nerve inhibition. This phenomenon refers to the binding of sodium channels by the anaesthetic agent, thus preventing the entry of sodium ions, which are essential for the transmission of nerve signals. The second mechanism suggested is related to the incidence of nerve fibre degeneration as a direct result of the pressure applied during the administration of intrapulpal anaesthesia [61]. The exact role of pressure, however, is yet to be fully understood.

While intrapulpal injections are typically done using an anaesthetic solution, some authors have stated that the technique is just as effective using a sterile saline solution, as long as the injection is performed under sufficient back pressure. This was explored in a double‐blinded procedure, where 56 patients reported inadequate numbness following the administration of a primary block or infiltration. Thirty‐seven patients received an intrapulpal injection comprising sterile saline, and 19 received one containing 2% lidocaine with 1 : 50,000 epinephrine. All injections, except three, where the pulpal exposure was too large to obtain sufficient pressure (two belonging to the lidocaine group and one to the saline group), were performed under back pressure. Results revealed complete, profound pulpal anaesthesia in all injections administered under back pressure [62].

Similar results were obtained during a later double‐blinded study wherein twenty‐three intrapulpal injections were administered, 14 of which contained 2% lidocaine with 1 : 100,000 epinephrine and 9 of which contained sterile saline. No statistically significant difference was found between the two groups, where 22 injections were deemed successful, indicating that back pressure plays a more significant role than the contents of the cartridge in the effectiveness of intrapulpal anaesthesia [60]. This, however, remains controversial due to the scarcity of research regarding the topic. Although existing evidence indicates that the success of intrapulpal anaesthesia largely depends on the back pressure generated rather than the anaesthetic solution itself, this conclusion is primarily based on limited and heterogeneous data. The translation of these findings to permanent teeth with vital pulps remains uncertain. It is therefore reasonable to consider that both mechanical and pharmacologic factors contribute to effective anaesthesia, and further high‐quality studies are warranted to delineate their respective roles.

The major advantage of intrapulpal anaesthesia is its efficiency in achieving profound pulpal anaesthesia in cases where other supplementary techniques have failed. Additionally, systemic effects associated with this technique are considered negligible [6]. On the other hand, disadvantages include the pain levels experienced by the patient during injection and the limited time of action. Moreover, this technique can only be utilized in the presence of pulpal exposure, unlike the other supplemental techniques.

To overcome one of the major disadvantages of intrapulpal anaesthesia, which is pain perception upon injection, Sooraparaju et al. [63] theorized that the topical application of a 20% benzocaine gel mixed with hyaluronidase to the pulp chamber could significantly reduce the incidence of pain during injection. Two hundred patients with mandibular first molars diagnosed with irreversible pulpitis, with a failed IANB, were randomly designated to two groups: one receiving 0.5 mL of 2% lignocaine as an intrapulpal injection and the other receiving the same but with prior placement of 20% benzocaine gel mixed with hyaluronidase. Pain perception of the control group was noted as ‘strong’, while that of the experimental group was reported to be ‘weak’ in nature, thus concluding a statistically significant difference in injection pain between both groups.

Another method of reducing pain perception during intrapulpal injection was explored. A total of one hundred patients who were subjected to failed IANB and intraligament injections using 2% lidocaine were assigned to the following groups: Group 27GN, where an intrapulpal injection using a 27‐gauge needle was administered; Group 27GT, where the same technique was carried out, in addition to a topical application of a lidocaine–prilocaine mixture; Group 31GN, where a 31‐gauge needle was used for the intrapulpal injection; and Group 31GT, where a combination of a 31‐gauge needle and a topical application of a lidocaine–prilocaine mixture were utilized. All intrapulpal injections were done using 0.2 mL or 2% lidocaine. The overall anaesthetic success of the intrapulpal injections was 100% for all groups. Additionally, both groups using a 31‐gauge needle exhibited significantly lower pain levels in comparison to those of the groups using a 27‐gauge needle. Topical anaesthesia application was found to lead to significant pain reduction when used with a 31‐gauge needle [64].

Whether or not the contents of the cartridge to be injected affect the anaesthetic success rate in mandibular permanent molars diagnosed with irreversible pulpitis is an area of research that still requires extensive investigation. The effect of different solutions on primary teeth with irreversible pulpitis, however, was explored on a sample of 40 patients. The solutions being compared in this study were bupivacaine, lignocaine, articaine and a sterile saline solution as a control group, all of which were performed under back pressure. Results failed to unveil a statistically significant difference between all four groups, all of which were successful in achieving sufficient pulpal anaesthesia, concluding that back pressure is the likely determining factor for intrapulpal anaesthesia rather than the solution used [65]. It is important to note that there is insufficient literature to support this claim, and additional research is required in this area, especially regarding permanent mandibular molars.

The current literature displays a gap regarding the topic of intrapulpal anaesthesia. While all the existing papers almost unanimously reported that the technique displays the maximum degree of pulpal anaesthesia, whether or not the contents of the cartridge play a role remains controversial. Additionally, there is an agreement that the procedure is linked with significant intraoperative pain, but overcoming this issue through the use of topical anaesthesia as well as larger gauge needles has been explored.

### 2.3. Cryoanaesthesia

Cryotherapy is yet another form of pain management that has been explored, with several ways, in which it can be combined with multiple anaesthetic techniques in order to reduce the incidence of intraoperative pain. This concept utilizes cold temperatures, which are known to induce vasoconstriction, as well as reduce oedema, which, in turn, leads to a significant reduction in the sensation of pain as well as cryogenic nerve suppression [66–70]. It was proposed earlier in endodontics as a way to decrease postoperative pain by utilizing cold irrigant solutions, after which its potential to decrease the incidence of intraoperative pain was explored [68, 70]. This concept can be applied either by cold application at the site of injection or intervention, or by cooling the anaesthetic solution itself. This will serve to reduce intraoperative pain, potentiating the anaesthetic effect and reducing the pain during injection.

The process of intraoperative cold application as a pain‐reducing technique was investigated. In this clinical trial, 60 patients diagnosed with symptomatic irreversible pulpitis received one of the following: a solitary IANB with 2% lignocaine, a combination of IANB with 2% lignocaine and buccal infiltration with 4% articaine or an IANB with 2% lignocaine in addition to cryotherapy. In the latter group, cryotherapy was utilized in the form of Endo‐Ice application before access opening, as well as ice stick placement postpulp exposure. Upon analysis of the results, it was revealed that the articaine group showed the most significant pain reduction during access cavity preparation, while both the articaine and cryotherapy groups exhibited the lowest pain levels during pulp extirpation, with no significant difference between the two. In addition, it was also noted that the cryotherapy group reported significantly reduced levels of patient anxiety [71].

Similarly, another study also aimed to observe the effect of Endo‐Ice application on the success rate of an IANB with 2% lidocaine, concerning one hundred and twenty‐six patients with irreversible pulpitis. As opposed to the aforementioned study’s results, the trial at hand showed a significant difference in pain reduction in the cryotherapy group compared to the control group [72].

An alternative method of cryotherapy application as an anaesthetic adjunct is through the intraoral application of ice packs on the vestibular surface of the tooth receiving treatment. This technique was performed by Topçuoğlu et al. [73], where one hundred and four patients with symptomatic irreversible pulpitis either received an IANB with 2% lidocaine or an IANB with 2% lidocaine in combination with intraoral ice‐pack application for 5 min postinjection. The success rate for the former was reported to be 30.8%, while the latter was 55.8%, with a statistically significant difference between the two. Therefore, it was concluded that this form of cryotherapy application could potentially significantly decrease the incidence of intraoperative pain.

In an attempt to determine the superior method of cryotherapy application, the difference in intraoperative pain reduction of different application techniques among 30 patients diagnosed with symptomatic irreversible pulpitis was observed. The patients were allocated to one of three groups, where they either received a standard IANB of 2% lignocaine, IANB in combination with intraoral vestibular ice‐pack application or IANB in combination with Endo‐Ice application. Statistical analysis revealed a significant difference in pain reduction only between the control and the Endo‐Ice groups. Additionally, IANB success rates were reported as 50% for the control group, 70% for the ice‐pack group and 80% for the Endo‐Ice group, with no existing significant difference between all groups [74].

In addition to the different techniques mentioned earlier, an innovative approach was investigated wherein the anaesthetic solution itself was cooled in order to serve as the cryotherapy application. This study comprised 60 patients with maxillary molars diagnosed with symptomatic irreversible pulpitis, who were randomly assigned to one of two groups: the control group, which received a buccal infiltration of 2% lidocaine, and the experimental group, which received the same infiltration, except the solution was cooled to 4°C–6°C. The results were in favour of the experimental group, where a success rate of 86.6% was observed, as opposed to that of the control group (26.6%). In addition, the cryoanaesthesia group also demonstrated a significant reduction in injection pain, as well as a significantly more rapid onset of anaesthesia [75]. The same concept was further studied, but in regard to the effect of cooling the anaesthetic solution on the success of an intraligamentary injection, rather than the initial IANB. This clinical trial included 88 patients with symptomatic irreversible pulpitis concerning lower molars, who had received a failed IANB of 2% lidocaine. Following the unsuccessful nerve block, the patients either received a standard intraligamentary injection of 2% lidocaine at room temperature or the same injection using a cartridge of 2% lidocaine, which has been refrigerated to a temperature of 4°C. The success rates reported were 59.1% and 52.7%, respectively, with no statistically significant difference, thus indicating that the temperature of the anaesthetic solution does not play a role in the level of anaesthetic efficiency [76].

The effect of cryotherapy application on anaesthetic efficiency has become an area of interest for research, particularly in regard to permanent molars. Elheeny et al. [77, 78], however, posed the question of whether age plays a role in the efficiency of cryotherapy as an anaesthetic adjunct. Two studies [77, 78] of a similar design were conducted by the aforementioned author, where the only variable was the age of the patients selected. In the first study [77], a total of one hundred and seventy children between the ages of 5 and 9 with primary molars diagnosed with symptomatic irreversible pulpitis were subjected to an IANB, where half received an additional intraoral ice‐pack application postinjection. The overall success rate of the group receiving cryotherapy was 79.2%, which was significantly higher than that of the control group, which exhibited a success rate of 50.6%. The second study [78], on the other hand, included one hundred and fifty‐two adolescents aged between 10 and 17, all of whom received an IANB of 4% articaine, following which only half were subjected to a 5‐min application of intraoral ice‐pack application in the buccal vestibule of the tooth to be treated. In agreement with the prior study’s results, there was a significant increase in the anaesthetic success rate in the cryotherapy group (59.2%), in comparison to the control group (40.8%). This was also confirmed by Fattahi et al. [79] in their randomized controlled trial. A recent study by Eldafrawi et al. also confirmed these results. Intrapulpal injection with a cooled cartridge at 4°C–5°C showed more profound anaesthesia compared to room temperature cartridge injection [6].

As a result of a dearth of articles regarding whether cryotherapy application is more effective through the use of intrapulpal ice sticks or Endo‐Ice, Gopakumar et al. [80] compared the following techniques and their success rates: a standard IANB of 2% lignocaine, IANB in combination with intrapulpal ice sticks, IANB in combination with Endo‐Ice and, finally, IANB in combination with both intrapulpal ice sticks and Endo‐Ice. This clinical trial consisted of a sample of two hundred patients with symptomatic irreversible pulpitis in relation to mandibular second molars, who were randomly allocated to one of the aforementioned groups. Statistical analysis deemed the difference significant, with success rates of 84% for the control group, 96% for the ice stick group, 92% for the Endo‐Ice group and 98% for the Endo‐Ice in combination with ice sticks. Alenezi et al. [81] in their recent systematic review with meta‐analysis confirmed that application of cryotherapy following IANB injection demonstrated higher success rates, significant reductions in both intraoperative and postoperative pain, and decreased dental anxiety levels.

While intraoperative pain is an area of concern in the field of endodontics, pain upon injection was evaluated. A sample of 90 patients was selected to receive one of the following: a buccal infiltration using 2% lidocaine, a topical application of 5% lidocaine gel preinfiltration or cryotherapy using ethyl chloride application at the anaesthetic site preinfiltration. It was reported that all patients in the control group experienced severe pain during injection, while 70% of the topical gel group reported moderate pain. As for the cryotherapy group, 46.67% experienced moderate pain and 50% experienced mild pain, thus concluding that cryotherapy is a more effective adjunct in comparison to topical gel application in terms of pain reduction during injection [82].

While the concept of cryoanaesthesia is still being explored, the available literature has highlighted its potential in significantly reducing the degree of intraoperative pain experienced by patients. This method is thought to be effective with both primary and supplementary anaesthetic techniques, and further research should be conducted in order to confirm this hypothesis.

There exists a clear lack of consensus on whether or not cooling the anaesthetic solution plays a role in increasing anaesthetic potency. This controversy is directly linked with the scarcity of literature in the field of cryoanaesthesia. Further trials should be carried out to investigate the relationship between anaesthetic success and a lower temperature of the solution used among various anaesthetic techniques. Additional research should also be conducted to close the large gap in evidence between intrapulpal anaesthesia compared to other supplemental techniques.

A limitation of the present narrative review is the inclusion of evidence derived from different anatomical contexts, including maxillary and mandibular teeth, primary and permanent dentition, and various tooth types, all of which are known to influence anaesthetic outcomes. While these anatomical differences may affect clinical applicability, the literature was intentionally discussed collectively to identify shared mechanistic themes related to inflammation, neural modulation and anaesthetic delivery. The findings should therefore be interpreted conceptually rather than as directly interchangeable clinical recommendations for specific tooth groups.

## 3. Conclusion

Effective pain management in mandibular molars with symptomatic irreversible pulpitis relies on a comprehensive strategy that combines optimized anaesthetic volume and supplemental techniques. Based on the synthesized evidence, a pragmatic, stepwise clinical approach may be considered when managing anaesthetic failure. Conventional nerve block anaesthesia should be regarded as the first‐line strategy, followed by supplemental techniques such as buccal infiltration or intraligamentary or intraosseous anaesthesia when profound pulpal anaesthesia is not achieved. Intrapulpal anaesthesia may be reserved as a final rescue technique in cases of persistent intraoperative pain. This escalation pathway is not intended as a rigid protocol but rather as a flexible clinical framework to support decision‐making, acknowledging that individual patient factors, tooth anatomy and inflammatory status ultimately guide anaesthetic selection. Future research should prioritize well‐designed, adequately powered randomized controlled clinical trials that directly compare stepwise anaesthetic strategies in teeth with symptomatic irreversible pulpitis, using standardized and clinically meaningful outcome measures. Particular emphasis is needed on elucidating the biological mechanisms underlying anaesthetic failure in inflamed pulps, including the role of sodium channel modulation, local tissue pH and neurogenic inflammation. Additionally, comparative effectiveness studies evaluating the timing and sequencing of supplemental anaesthetic techniques, as well as patient‐centred outcomes, such as intraoperative pain perception and anxiety, are warranted. Establishing consensus definitions for anaesthetic success and failure would further strengthen the evidence base and improve comparability across future investigations.

## Funding

No funding was received for this manuscript.

## Conflicts of Interest

The authors declare no conflicts of interest.

## Data Availability

The data that support the findings of this study are available from the corresponding author upon reasonable request.
